# Supplementary material for: The interactome of intact mitochondria by cross-linking mass spectrometry provides evidence for coexisting respiratory supercomplexes
Source: Mol Cell Proteomics. 2017 Dec 8;17(2):216–32. doi: 10.1074/mcp.RA117.000470 (PMC5795388; doi:10.1074/mcp.RA117.000470)
Supplement: Supplemental Data [file supp_RA117.000470_133922_0_supp_23978_fzffwf.zip › spectra_annotation/mito_DR_spectra_annotation/62-1-3-1-1-1.pdf]

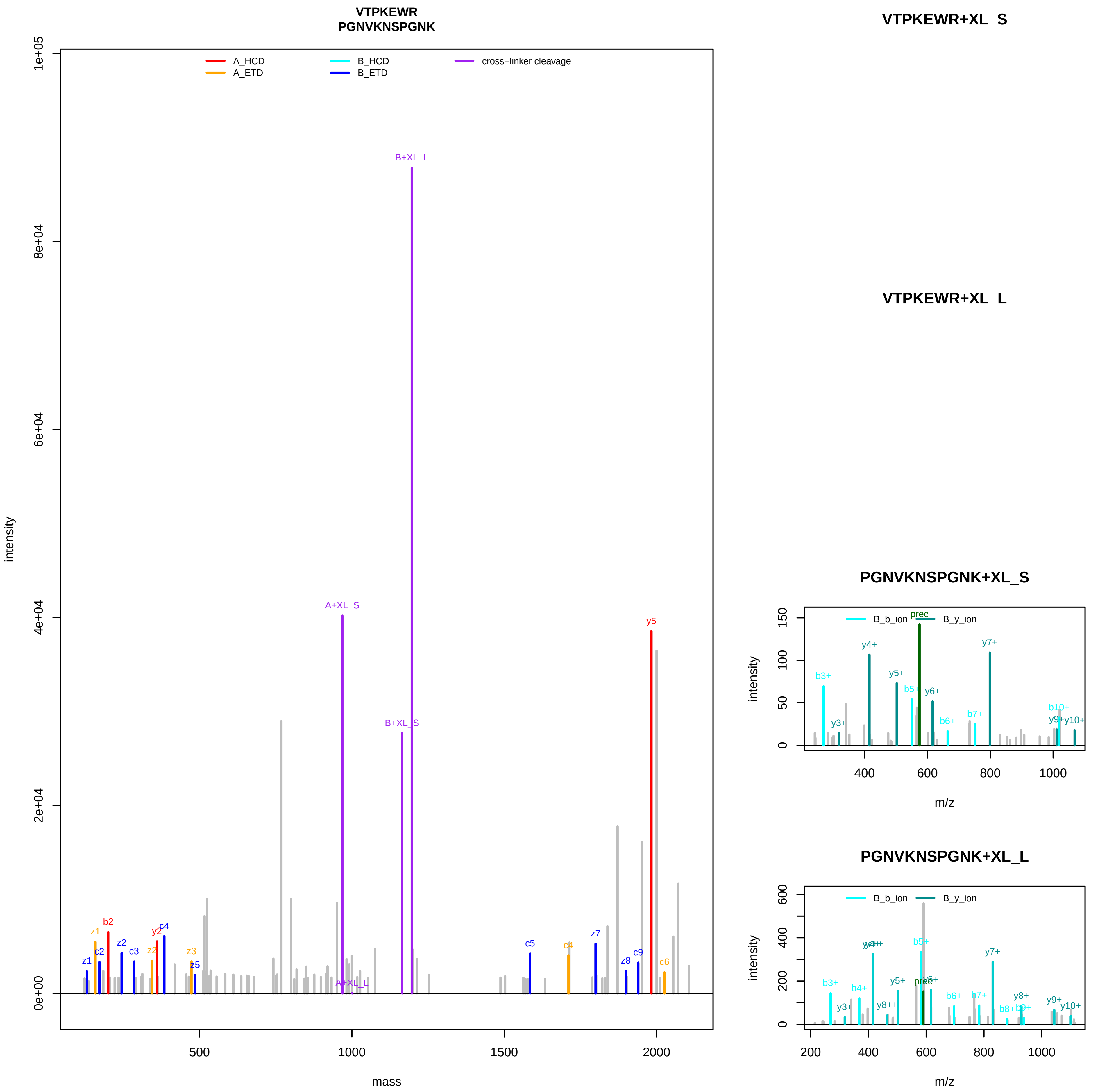

VTPKEWR
PGNVKNSPGNK
A_HCD
A_ETD
B_HCD
B_ETD
cross−linker cleavage
B+XL_L
A+XL_S
B+XL_S
A+XL_L
y5
b2
y2
z1
c2
z2
c3
z2
c4
z3
z5
c5
c4
z7
z8
c9
c6
intensity
mass
0e+00
2e+04
4e+04
6e+04
8e+04
1e+05
500
1000
1500
2000
VTPKEWR+XL_S
VTPKEWR+XL_L
PGNVKNSPGNK+XL_S
B_b_ion
B_y_ion
prec
b3+
y3+
y4+
y5+
b5+
y6+
b6+
b7+
y7+
b10+
y9+
y10+
intensity
m/z
0
50
100
150
400
600
800
1000
PGNVKNSPGNK+XL_L
B_b_ion
B_y_ion
b3+
y3+
b4+
y4++
y8++
y5+
b5+
prec
y6+
b6+
b7+
y7+
b8+
y8+
b9+
y9+
y10+
intensity
m/z
0
200
400
600
200
400
600
800
1000
